# Comparison of exercise, dobutamine-atropine and dipyridamole-atropine stress echocardiography in detecting coronary artery disease

**DOI:** 10.1186/1476-7120-4-22

**Published:** 2006-05-03

**Authors:** Ivana Nedeljkovic, Miodrag Ostojic, Branko Beleslin, Ana Djordjevic-Dikic, Jelena Stepanovic, Milan Nedeljkovic, Sinisa Stojkovic, Goran Stankovic, Jovica Saponjski, Zorica Petrasinovic, Vojislav Giga, Predrag Mitrovic

**Affiliations:** 1 University Institute for Cardiovascular Diseases, Department for Diagnostic and Catheterization Laboratories, Clinical Center of Serbia, Serbia and Montenegro

## Abstract

**Background:**

Dipyridamole and dobutamine stress echocardiography testing are most widely utilized, but their sensitivity remained suboptimal in comparison to routine exercise stress echocardiography. The aim of our study is to compare, head-to-head, exercise, dobutamine and dipyridamole stress echocardiography tests, performed with state-of-the-art protocols in a large scale prospective group of patients.

**Methods:**

Dipyridamole-atropine (Dipatro: 0.84 mg/kg over 10 min i.v. dipyridamole with addition of up to 1 mg of atropine), dobutamine-atropine (Dobatro: up to 40 mcg/kg/min i.v. dobutamine with addition of up to 1 mg of atropine) and exercise (Ex, Bruce) were performed in 166 pts. Of them, 117 pts without resting wall motion abnormalities were enrolled in study (91 male; mean age 54 ± 10 years; previous non-transmural myocardial infarction in 32 pts, angina pectoris in 69 pts and atypical chest pain in 16 pts). Tests were performed in random sequence, in 3 different days, within 5 day period under identical therapy. All patients underwent coronary angiography.

**Results:**

Significant coronary artery disease (CAD; ≥50% diameter stenosis) was present in 69 pts (57 pts 1-vessel CAD, 12 multivessel CAD) and absent in 48 pts. Sensitivity (Sn) was 96%, 93% and 90%, whereas specificity (Sp) was 92%, 92% and 87% for Dobatro, Dipatro and Ex, respectively (p = ns). Concomitant beta blocker therapy did not influence peak rate-pressure product and Sn of Dobatro and Dipatro (p = ns).

**Conclusion:**

When state-of-the-art protocols are used, dipyridamole and dobutamine stress echocardiography have comparable and high diagnostic accuracy, similar to maximal post-exercise treadmill stress echocardiography.

## Background

Exercise stress echocardiography is more sensitive and specific for detecting inducible ischemia than exercise electrocardiography testing alone [1-4]. Dipyridamole and dobutamine stress echocardiography testing are most widely utilized, but their sensitivity remained suboptimal in comparison to routine exercise stress echocardiography [5,6]. This diagnostic challenge provoked development of stress protocols including addition of atropine [7-12]. The objective of this study was to assess in head-to-head fashion diagnostic value of dipyridamole-atropine, dobutamine-atropine and exercise stress echocardiography in the same group of patients presented for evaluation of coronary artery disease.

## Methods

### Study population

Between January and July 2004, 166 consecutive patients referred for coronary angiography were evaluated. Of them, only 117 (91 male, 26 female; mean age 54 ± 10 years) patients without resting wall motion abnormalities were enrolled in the study. Exclusion criteria were: presence of left ventricular wall motion abnormality at baseline, heart failure, left bundle branch block, unstable angina, congenital or valvular heart disease, severe hypertension (systolic ≥180 mmHg and diastolic pressure ≥110 mmHg), serious arrhythmias and chronic obstructive pulmonary disease.

Informed consent was obtained from all patients. They underwent exercise, and pharmacological stress echocardiography. Previous non-transmural myocardial infarction was present in 32 patients, 69 had angina pectoris and 16 patients experienced atypical chest pain.

The study was approved by the Institutional Review Board of the Institute of Cardiovascular Diseases in Belgrade.

Concomitant beta blockers were used in 34% (40/117), calcium antagonists in 37% (44/117) and nitrates in 45% (53/117) of patients. Teophylline, caffeine-containing products, and dipyridamole preparations were not allowed for at least 12 hours before testing.

Patients performed stress testing in 3 different days in random sequence within 5 day period, at least 14 days after uncomplicated myocardial infarction.

### Stress protocols

*Exercise echocardiography *(*Ex*) was performed according to maximal Bruce treadmill protocol.

#### Dobutamine-atropine (Dobatro)

Dobutamine was infused in 3-minutes dose increments, starting from 5 to 40 mcg/kg/min. In echocardiography negative patients, atropine was added (in 4 divided doses up to a maximum of 1 mg of atropine) to the continuing 40 mcg/kg/min dobutamine infusion [11].

#### Dipyridamole-atropine (Dipatro)

Dipyridamole was infused at a dose of 0.56 mg/kg over 4 min, followed with 4 min of no dose and then, if the test was still negative, 0.28 mg/kg in 2 min. In dipyridamole echocardiography negative patients, 3 min after the end of infusion, atropine was given in 4 divided doses up to a maximum of 1 mg of atropine [12].

The test was considered positive in the presence of obvious left ventricular regional wall motion abnormality. The other reasons for test interruption were: peak atropine dose (for pharmacological tests), achievement of maximal age predicted heart rate, significant ST segment depression or elevation, severe chest pain, exercise-limiting dyspnea, fatigue and/or claudication, symptomatic hypotension (decrease in systolic blood pressure >20 mmHg) or hypertension (>220/120 mmHg), severe arrhythmias or intolerable side effects of administered drugs. Intravenous aminophylline (250 mg) was given after cessation of Dipatro test, and beta blockers (metoprolol 5 mg) or nitroglycerin if required.

A 12-lead electrocardiogram monitoring was performed continuously and recorded at baseline, at the end of each stage and during recovery period accompanied with blood pressure recordings. Rate pressure product was calculated by multiplying systolic blood pressure and heart rate.

### Echocardiographic analysis

Two-dimensional echocardiography was performed with the patient in the left lateral decubitus position. Standard apical and parasternal views were recorded, facilitating the analysis from the off line digitized videotapes (Image View, ATL). We used 16-segment left ventricular model [13]. Segmental wall motion was evaluated using standard method: normal – 1, hypokinetic – 2, akinetic – 3, or dyskinetic – 4 [13]. Wall motion score index was derived for rest and peak stress tests. Video tapes were analyzed independently by two experienced observers unaware of patients' data or other tests results with overall agreement of 92%. By subgroup analysis, the interobserver agreement was 93%, 94% and 90% for Dobatro, Dipatro, and Ex. In case of discrepancy decision was made by consensus.

### Coronary angiography and quantitative angiographic analysis

All patients underwent selective coronary angiography according to Judkin's technique, within one week of stress echocardiography tests. Angiograms were analyzed using quantitative coronary angiography (MEDIS CMS, Leiden, The Netherlands) by observers unaware of the patient's data. Significant coronary artery stenosis was considered as ≥50% diameter stenosis present in at least one major epicardial coronary vessel.

### Statistical analysis

The data are expressed as mean ± SD. Comparison of continuous variables was performed using ANOVA As, Newman-Keuls procedure and *t *test where appropriate, whereas dichotomous variables were compared using chi-square (McNemar-s test for paired proportions). A coefficient of correlation (r) was used to compare peak wall motion score index of different tests. Confidence intervals were calculated according to standard formulas (95%CI) as well as sensitivity, specificity and diagnostic accuracy.

Calculation of sensitivity, specificity and diagnostic accuracy were performed according to standard formulas. A p value less than 0.05 was considered statistically significant.

## Results

### Angiographic characteristics

Coronary artery disease was present in 69 patients: one-vessel coronary artery disease was present in 57 patients, 12 patients had multi-vessel coronary artery disease. The distribution of lesions were: left anterior descending – 50 patients, circumflex artery – 15 patients, and right coronary artery – 16 patients.

### Feasibility, safety and hemodynamic changes

Feasibility was 95% and 97% for Dobatro and Dipatro (p = ns), respectively. Limiting side effects occurred in 6 and in 4 patients during Dobatro and Dipatro, including non-sustained ventricular tachycardia and short run of supraventricular tachycardia in the absence of myocardial ischemia. They disappeared after cessation of the test or after administration of specific antidote.

Limiting side effects occurred in 17 patients (14%) during Ex in the absence of diagnostic end point and consisted of serious ventricular and supraventricular rhythm disturbances, severe chest pain, hypertensive response and fatigue. Thus, feasibility of Ex was 85%. There was no significant difference in feasibility of all three tests (p = ns for all intergroup differences). There were no late complications in the ensuing hours after finishing the tests.

Hemodynamic changes during stress echocardiography tests are presented in Table 1.

**Table 1:** Peak hemodynamic data during dobutamine, dobutamine-atropine, dipyridamole, dipyridamole-atropine and exercise stress tests.

	Dob	Dobatro	Dip	Dipatro	Ex
Heart rate, beat/min	110 ± 27	138 ± 25*	91 ± 15	125 ± 25*	148 ± 22**
Systolic blood pressure, mmHg	155 ± 20	160 ± 30*	138 ± 22	158 ± 20*	182 ± 24**
Diastolic blood pressure, mmHg	98 ± 15	103 ± 10	90 ± 9	97 ± 12	109 ± 14**
PRR, mmHg × beat/min/100	170 ± 64	221 ± 60*	126 ± 32	198 ± 59*	348 ± 61**

Values are given as mean ± SD. Dob indicates dobutamine; Dobatro, dobutamine-atropine; Dip, dipyridamole; and Dipatro, dipyridamole-atropine; RPP, rate-pressure product. Asterisk indicates significant difference for Dobatro vs. Dob, and Dipatro vs. Dip (p < 0.0001). Two asterisks indicate significant difference between Ex and other tests (p < 0.0001 for all).

### Diagnostic value of stress echocardiography

**Figure 1:**
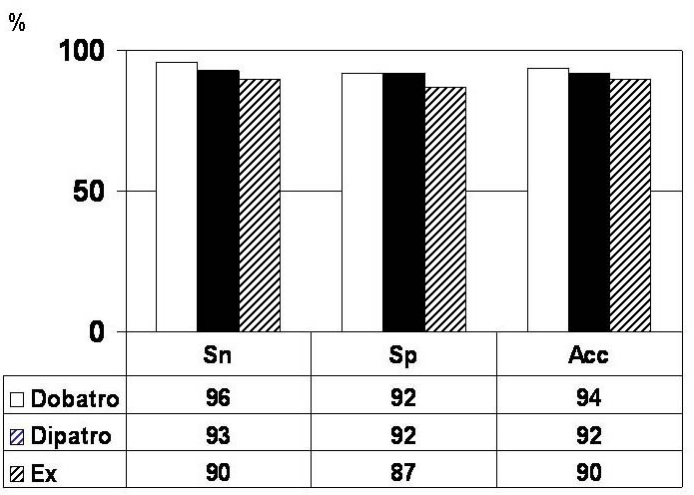
**Sensitivity, specificity and diagnostic accuracy of stress echocardiography tests**. Bar graph showing sensitivity (Sn), specificity (Sp) and diagnostic accuracy (Acc) of dobutamine-atropine (Dobatro), dipyridamole-atropine (Dipatro) and exercise (Ex) stress echocardiography tests. There was no statistically significant difference between three tests. Asterisk indicates significant difference between stress tests (p < 0.01).

Atropine was added to dobutamine in 69% (81/117) of patients and to dipyridamole in 68% (80/117) of patients. Stress-induced wall motion abnormalities appeared in 70, 68 and 68 patients during Dobatro, Dipatro and Ex, respectively. The sensitivity was 96%, 93% and 90% for Dobatro, Dipatro and Ex in detection of myocardial ischemia (p = ns for Ex vs. Dobatro vs. Dipatro) (Figure 1). Specificity was 92% both for Dobatro and Dipatro, and 87% for Ex (p = ns for all intergroup differences). Diagnostic accuracy was: 94% for Dobatro, 92% for Dipatro and 90% for Ex, respectively (p = ns for Ex vs. Dobatro vs. Dipatro).

Dobatro, Dipatro and Ex provoked significant change from the rest to peak stress WMSI (1.32 ± 0.18, 1.31 ± 0.17 and 1.28 ± 0.18, for Dobatro, Dipatro, and Ex; p = ns for all), with significant correlation (p < 0.0001) of peak WMSI between all tests.

Single vs. multivessel CAD: The sensitivity of stress in detection of one-vessel coronary artery disease was 95% for Dobatro and 95% for Dipatro and 93% for Ex (p = ns for Ex vs. Dobatro, and Ex vs. Dipatro). The sensitivity for detection of multivessel coronary artery disease was 100% for Dobatro and Dipatro and 92% for Ex (p = ns).

### The impact of concomitant beta – blocker therapy on stress echocardiographic results

Forty (34%) patients received concomitant beta blocker therapy (34 with coronary artery disease). There was significant difference between patients with (BB+) and without beta-blocker therapy (BB-) in the peak heart rate for Dob, Dip, and Ex (p < 0.001), whereas addition of atropine excluded significant influence of beta-blocker therapy on peak heart rate. Rate-pressure product at baseline and peak stress tests in patients with (BB+) and without (BB-) concomitant beta-blocker therapy are presented in Table 2.

**Table 2:** Rate-pressure product at baseline and peak stress tests in patients with (BB+) and without (BB-) concomitant beta-blocker therapy

RPP (mmHgxbpm/100)	baseline	Dob	Dobatro	Dip	Dipatro	Ex
BB+ group	110 ± 25	167 ± 27	213 ± 58	135 ± 10	199 ± 37	233 ± 22
BB- group	123 ± 40*	205 ± 20	230 ± 53	145 ± 12*	210 ± 40	389 ± 77*

RPP indicates rate-pressure product; Dob indicates dobutamine; Dobatro, dobutamine-atropine; Dip, dipyridamole; Dipatro, dipyridamole-atropine; Values are given as mean ± SD. Asterisk indicates significant difference between stress tests (p < 0.001).

**Figure 2:**
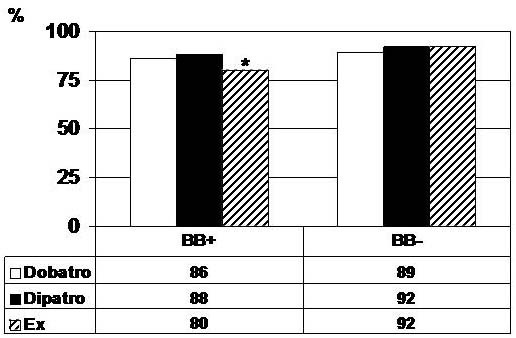
**Sensitivity of stress echocardiography tests in patients with and without concomitant beta-blocker therapy**. Sensitivity of dobutamine-atropine (Dobatro), dipyridamole-atropine (Dipatro) and exercise (Ex) in patients with and without concomitant beta-blocker therapy. There was no significant difference in sensitivity for Dobatro and Dipatro between patients with (BB+) and without (BB-) concomitant beta-blocker therapy, whereas sensitivity of Ex was significantly higher in patients without concomitant beta-blocker therapy. Asterisk indicates significant difference between stress tests (p < 0.01).

Atropine was added to dobutamine in 75% of patients in BB+ (34/40) and in 61% in BB- group (47/77), and to dipyridamole in 85% of patients in BB+ (34/40) and 60% (46/77) in BB- group. Addition of atropine resulted in similar sensitivity (Dobatro: BB+ 86% vs. BB- 89%, p = ns; Dipatro: BB+ 88% vs. BB- 92%, p = ns). However, sensitivity of Ex was significantly affected by beta-blocker therapy (BB+ 80% vs. BB- 92%, p < 0.01) (Figure 2).

## Discussion

This study represents for the first time direct comparative evaluation of dobutamine-atropine and dipyridamole-atropine – with exercise stress echocardiography performed in the same group of patients. Dipyridamole-atropine and dobutamine-atropine stress echocardiography have comparable and high diagnostic accuracy, similar to maximal post-exercise treadmill stress echocardiography. In addition, dipyridamole-atropine and dobutamine-atropine stress echocardiography testing overcomes the effects of concomitant beta-blocker therapy reaching high and comparable diagnostic value.

Our results are comparable with previous findings showing that atropine coadminitsration significantly improved sensitivity in dobutamine and dipyridamole negative patients [11,12]. In addition, Pingitore et al. have shown, in comparative study with dobutamine-atropine and dipyridamole-atropine, that both tests have comparative sensitivity of 84% and 82%, respectively, without significant difference between them [14].

It has been shown that beta-blocker therapy can significantly influence results of stress echocardiography tests if routine doses are employed. Beta-blockers are known to protect from exercise induced ischemia [15]. They also affect the results of dipyridamole stress echocardiography, despite the fact that hemodynamic profile is at least affected by dipyridamole [16]. In our study, addition of atropine induced significant increase in heart rate, systolic blood pressure and rate pressure product as well as increase of diagnostic accuracy in comparison to dobutamine and dipyridamole alone regardless of beta blocker therapy. In comparison to exercise stress echocardiography testing, atropine coadministration resulted in similar sensitivity and specificity of dobutamine-atropine and dipyridamole-atropine stress echocardiography in both groups of patients. Thus, atropine factor in pharmacological stress echocardiography testing can overcome the effects of beta-blocker therapy, as it has been shown in previous studies [17,18].

### Pathophysiological mechanisms

We used three tests with different mechanisms of provoking myocardial ischemia through a) an increase in oxygen demand, exceeding the fixed supply – dobutamine and exercise; and b) flow maldistribution, due to inappropriate coronary artery vasodilatation. Atropine superimposes a marked chronotropic stress to dipyridamole and dobutamine increasing oxygen demand, decreasing, at the same time, myocardial oxygen supply by shortening the diastole whose duration is important for perfusion in the presence of maximal vasodilatation [19] and increasing the ischemic potential of stress echocardiography [11,12].

### Comparison with previous studies

Several meta-analytic comparisons of echocardiographic stressors have been performed in the past [20-23]. They unanimously reached the conclusion that dipyridamole is more specific than dobutamine and exercise, and exercise and dobutamine are more sensitive than dipyridamole for noninvasive detection of coronary artery disease affecting the recent cardiology guidelines [4] and standard textbook knowledge [24], which suggests to use dipyridamole stress in combination with perfusion scintigraphy and consider only dobutamine stress as a suitable pharmacological stress to be combined with echocardiography. However, this conclusion conflicts the results of present study. The reason of this apparent discrepancy is the fact that with the vasodilator stress echocardiography, one needs the high dose protocol with atropine to optimize the diagnostic sensitivity. The same result can be obtained, without atropine, by using the high dose over a shorter infusion time of dipyridamole: the so called accelerated protocol [25]. As a matter of fact, the 1998. Guidelines of the American Society of Echocardiography clearly recommended high dose + atropine, as the standard protocol to achieve optimal accuracy with dipyridamole stress echocardiography [13]. Accordingly, if we consider only the literature with state-of-the-art dipyridamole protocols (with accelerated infusion or with atropine coadministration), the conclusions of the guidelines and recent textbook recommendations clash against available evidence. Two previous reports [26,27] comparing accelerated dipyridamole versus dobutamine stress echocardiography tests, and 2 additional reports [14,28] comparing dipyridamole-atropine stress echocardiography versus dobutamine stress echocardiography, have reached consistent conclusion of each individual study – cumulative analysis has shown that dipyridamole had a better specificity and the same sensitivity in comparison with high dose dobutamine stress echo. These results of the published literature, as well as the results of the present study, represent a weight of evidence which may influence current guidelines and recent cardiology textbooks statements. When state of the art protocols are used, either dobutamine or dipyridamole provide excellent and comparable diagnostic sensitivity and overall accuracy.

### Study limitations

The study group was derived from patients referred for coronary angiography and angioplasty, so large majority of patients had the one-vessel coronary artery disease. The use of a qualitative assessment of wall motion during stress echocardiography is a limitation of this technique, although qualitative assessment of regional wall motion by trained observers remains the only clinically applied method in stress echocardiography.

## Conclusion

When state-of-the-art protocols are used, dipyridamole and dobutamine stress echocardiography have comparable and high diagnostic accuracy, similar to maximal post-exercise treadmill stress echocardiography. In addition, dipyridamole-atropine and dobutamine-atropine stress echocardiography testing overcome the effects of concomitant beta-blocker therapy reaching high and comparable diagnostic value.

## Competing interests

The author(s) declare that they have no competing interests.

## Authors' contributions

We would like to report specific contribution of each author of the manuscript: IN made the concept, performed stress echocardiography tests and participated in the echocardiographic analysis. MO participated in the design of the study and interpretation of data. BB performed quantitative coronary angiography and help to draft the manuscript. ADD carried out the stress echocardiography testing. JS performed stress echocardiography tests and participated in its interpretation. MN performed coronary angiography. SS performed coronary angiography and quantitative coronary angiography analysis. GS participated in the interpretation of data. JS helped in quantitative coronary angiographic analysis. ZP carried out the selection of patients. VG participated in the statistic analysis. PM participated in the design of study and helped to draft the manuscript.

All authors read and approved the final manuscript.

## References

[B1] Marwick T (2003). Stress echocardiography. Heart.

[B2] Marwick T, Nemec J, Pashow F, Stewart WJ, Salcedo EE (1992). Accuracy and limitation of routine exercise echocardiography in a routine clinical setting. J Am Coll Cardiol.

[B3] Ryan T, Vasey CG, Presti CF, O'Donnell JA, Feigenbaum H, Armstrong WF (1988). Exercise echocardiography: Detection of coronary artery disease in patients with normal left ventricular wall motion at rest. J Am Coll Cardiol.

[B4] Cheitlin MD, Armstrong WF, Aurigemma GP, Beller GE, Bierman FZ, Davis JL, Douglas PS, Faxon DP, Gillam LD, Kimball TR, Kussmaul WG, Pearlman AS, Philbrick JT, Rakowski H, Thys DM, Antman EM, Smith SC, Alpert JS, Gregoratos G, Anderson JL, Hiratzka LF, Faxon DP, Hunt SA, Fuster V, Jacobs AK, Gibbons RJ, Russell RO (2003). ACC/AHA/ASE 2003 guideline update for the clinical application of echocardiography: Summary article. A report of the American College of Cardiology/American Heart Association Task Force on Practice Guidelines (ACC/AHA/ASE Committee to Update the 1997 Guidelines on the Clinical Application of Echocardiography). J Am Coll Cardiol.

[B5] Picano E, Distante A, Masini M, Morales MA, Lattanzi F, L'Abbate A (1985). Dipyridamole echocardiography test in effort angina pectoris. J Am Coll Cardiol.

[B6] Salustri A, Fioretti PM, Pozzoli NMA, McNeil AJ, Roelandt JR (1992). Dobutamine stress echocardiography: its role in the diagnosis of coronary artery disease. Eur Heart J.

[B7] Picano E, Lattanzi F, Masini M, Distante A, L'Abbate A (1986). High dose dipyridamole echocardiography test in effort angina pectoris. J Am Coll Cardiol.

[B8] Ling LH, Pellikka PA, Mahoney DW, Oh JK, McCully RB, Roger VL, Seward JB (1996). Atropine augmentation in dobutamine stress echocardiography: role and incremental value in a clinical practice setting. J Am Coll Cardiol.

[B9] Beleslin BD, Ostojic M, Stepanovic J, Djordjevic-Dikic A, Stojkovic S, Nedeljkovic M, Stankovic G, Petrasinovic Z, Gojkovic Lj, Vasiljevic-Pokrajcic Z (1994). Stress echocardiography in the diagnosis of ischemic heart disease: head-to head comparison between exercise, dobutamine and dipyridamole tests. Circulation.

[B10] Ostojic M, Picano E, Beleslin B, Dordjevic-Dikic A, Distante A, Stepanovic J, Reisenhofer B, Babic R, Stojkovic S, Nedeljkovic M (1994). Dipyridamole-dobutamine echocardiography: a novel test for detection of milder forms of coronary artery disease. J Am Coll Cardiol.

[B11] McNeil AJ, Fioretti PM, el-Said SM, Distante A, Stepanovic J, Reisenhofer B, Babic R, Stojkovic S, Nedeljkovic M (1992). Enhanced sensitivity for detection of coronary artery disease by addition of atropine to dobutamine stress echocardiography. Am J Cardiol.

[B12] Picano E, Pingitore A, Conti U, Kozakova M, Boem A, Cabani E, Cuiti M, Distante A, L'Abbate A (1993). Enhanced sensitivity for detection of coronary artery disease by addition of atropine to dipyridamole echocardiography. Eur Heart J.

[B13] Armstrong WF, Pellikka PA, Ryan T, Crouse L, Zoghbi WA (1998). Stress echocardiography: recommendations for performance and interpretation of stress echocardiography. Stress Echocardiography Task Force of the Nomenclature and Standards Committee of the American Society of Echocardiography. J Am Soc Echocardiograph.

[B14] Pingitore A, Picano E, Colosso MQ, Reisenhofer B, Gigli G, Lucarini AR, Petix N, Previtali M, Bigi R, Chiaranda G, Minardi G, de Alcantara M, Lowenstein J, Sclavo MG, Palmieri C, Galati A, Seveso G, Heyman J, Mathias W, Casazza F, Sicari R, Raciti M, Landi P, Marzilli M (1996). The atropine factor in pharmacologic stress echocardiography. Echo Persantine (EPIC) and Echo Dobutamine International Cooperative (EDIC) Study Groups. J Am Coll Cardiol.

[B15] Ross J (1989). Mechanisms of regional ischemia and antianginal drug action during exercise. Prog Cardiovasc Dis.

[B16] Ferrara N, Longobardi G, Nicolino A, Acanfora D, Odierna L, Furgi G, Rossi M, Leosco D, Rengo F (1992). Effect of beta-adrenoceptor blockade on dipyridamole induced myocardial asynergies in coronary artery disease. J Am Coll Cardiol.

[B17] Fioretti P, Polderman D, Salustri A, Forster T, Belloti P, Boersma E, McNeill AI, el-Said ES, Roelandt JR (1994). Atropine increases the accuracy of dobutamine stress echocardiography in patients taking beta-blockers. Eur Heart J.

[B18] Lattanzi F, Picano E, Bolognese L, Piccinino C, Sarasso G, Orlandini A, L'Abbate A (1991). Inhibition of dipyridamole-induced ischemia by antianginal therapy in humans: Correlation with exercise electrocardiography. Circulation.

[B19] Bache RJ, Cobb FR (1977). Effect of maximal coronary vasodilation on transm, ural perfusion during tachycardia in awake dog. Circ Res.

[B20] Kim C, Kwok YS, Heagerty P, Redberg R (2001). Pharmacologic stress testing for coronary disease diagnosis: A meta-analysis. Am Heart J.

[B21] Noguchi Y, Nagata-Kobayashi S, Stahl JE, Wong JB (2005). A meta-analytic comparison of Echocardiographic stressors. Intl J Cardiovasc Imaging.

[B22] Albuquerque Fonseca L, Picano E (2001). Comparison of dipyridamole and exercise stress echocardiography in detection of coronary artery disease (A meta-analytsis). Am J Cardiol.

[B23] Picano E, Bedetti G, Varga A, Cseh E (2000). The comparable diagnostic accuracies of dobutamine-stress and dipyridamole-stress echocardiographies: a meta-analysis. Coronary Artery Disease.

[B24] Beller GA, Zipes DP, Libby P, Bonow RO, Braunwald E (2005). Relative merits of cardiac diagnostic techniques. Heart disease A textbook of cardiovascular medicine.

[B25] Dal Porto R, Faletra F, Picano E, Pirelli S, Moreo A, Varga A (2001). Safety, feasibility and diagnostic accuracy of accelerated high-dose stress echocardiography. Am J Cardiol.

[B26] Salustri A, Fioretti PM, McNeill AJ, Pozzoli MM, Roelandt JR (1992). Pharmacological stress echocardiography in the diagnosis of coronary artery disease and myocardial ischemia: a comparison between dobutamine and dipyridamole. Eur Heart J.

[B27] San Roman JA, Vilacosta I, Castillo JA, Rollan MJ, Hernandez M, Peral V, Garcimartin I, de la Torre MM, Fernandez-Aviles F (1998). Selection of the optimal stress test for the diagnosis of coronary artery disease. Heart.

[B28] Loimaala A, Groundstroem K, Pasanen M, Oja P, Vuori I (1999). Comparison of bicycle, heavy isometric, dipyridamole-atropine and dobutamine stress echocardiography for diagnosis of myocardial ischemia. Am J Cardiol.

